# Dietary variables associated with substantial postpartum weight retention at 1-year among women with GDM pregnancy

**DOI:** 10.1186/s40608-017-0166-0

**Published:** 2017-08-03

**Authors:** Jaimie N. Davis, Grace E. Shearrer, Wei Tao, Shanta R. Hurston, Erica P. Gunderson

**Affiliations:** 1 Department of Nutritional Sciences, University of Texas at Austin, 103 24 street, Building PAI 3.24, Austin, TX 78712 USA; 2 Kaiser Permanente Northern California, Division of Research, Oakland, California USA

**Keywords:** Soda, Dietary intake, Postpartum weight retention, Gestational diabetes

## Abstract

**Background:**

An understanding of the dietary behaviors linked to substantial postpartum weight retention, particularly in women diagnosed with gestational diabetes (GDM), is warranted to focus intervention efforts to prevent future type 2 diabetes. This study evaluates the relationship between dietary food intake at 6–9 weeks postpartum (baseline) and odds of substantial postpartum weight retention (≥ 5 kg) at 1-year in women with GDM.

**Methods:**

The Study of Women, Infant Feeding and Type 2 Diabetes after GDM pregnancy (SWIFT) is a prospective multi-ethnic cohort (75% minority) of 1035 women (aged 20–45 years) with recent GDM who delivered a singleton, live birth (≥35 weeks gestation) and underwent 2-h 75 g OGTTs, anthropometric measurements and other assessments at 6–9 weeks postpartum (baseline) and annually thereafter. Eight hundred and eighty-eight women without diabetes at baseline completed the 18-item PrimeScreen to assess dietary intake and the 13-item Caffeine Survey to assess beverage intake, and completed 1-year follow-up. Average postpartum weight retention was calculated (1-year postpartum weight minus pre-pregnancy weight). Multivariable logistic regression models that estimated baseline dietary intake and odds of substantial postpartum weight retention (SPPWR ≥5 kg above pre-pregnancy weight) versus not SPPWR adjusted for numerous clinical, sociodemographic and behavioral covariates.

**Results:**

Compared to eating no fried foods, women who reported eating fried foods ≥5 servings/wk. (*n* = 32) and 2–4 serv/wk. (*n* = 208), respectively, had a three-fold and two-fold higher odds of SPPWR (OR = 3.38, 95% CI:1.36–8.38, *P* = 0.009; OR = 1.99, 95% CI:1.30–3.03, *P* = 0.02), after adjustment for covariates and other foods and soda intake. Soda intake ≥2 serv/wk. versus none was associated with higher odds of SPPWR (OR = 1.95, 95% CI:1.22–3.11, *P* = 0.005) adjusted for fried foods and covariates, but was attenuated (OR = 1.61,95% CI:0.98–2.66, *p* = 0.06) after addition of whole eggs and processed meats.

**Conclusions:**

These findings indicate that interventions should focus on reducing fried foods and soda intake during early postpartum periods to reduce substantial postpartum weight retention in high-risk women with GDM.

**Trial registration:**

NCT01967030; October 2013, Eunice Kennedy Shriver National Institute of Health and Human Development (NICHD).

## Background

Gestational diabetes mellitus (GDM), defined as glucose intolerance with onset or first recognition during pregnancy, is one of the most common complications in pregnancy, representing about 19% of pregnancies annually, using the new International Association of the Diabetes and Pregnancy Study Groups (IADPSG) criteria [1]. Being overweight or obese before pregnancy, as well as excessive gestational weight gain (GWG), are leading risk factors for developing GDM [2, 3]. Women with GDM compared to women with normoglycemic pregnancies are seven times more likely to develop type 2 diabetes (T2D) later in life [4]. Postpartum weight change appears to influence future risk of T2D in women with a history of GDM. In more than 600 Hispanic women with GDM, a postpartum weight gain of 4.5 kg was independently associated with a twofold increase in the risk of developing diabetes [5]. In a prospective cohort study with up to 18 years of follow-up among 1695 primarily Caucasian GDM women, each 5 kg increment of weight gain after GDM was associated with a 27% higher risk of developing T2D [6]. Pregnancy weight retention can vary greatly, with 20% of women in general having substantial postpartum weight retention (SPPWR), defined as ≥5 kg at 1 year postpartum [7], and women with GWG above recommendations retaining the most weight [8].

Identifying dietary predictors of SPPWR, particularly in GDM women, would focus behavioral intervention efforts for weight reduction. A large national retrospective study showed that women with GDM compared to their counterparts without GDM were significantly less likely to meet the five or more servings of fruit and vegetable recommendations [9]. Recently, there have been a handful of randomized controlled trials targeting postpartum weight reduction in GDM women [10]. A lifestyle intervention for 397 postpartum women (45% African American) who were overweight/obese reported that lower junk food at six weeks postpartum was associated with greater weight loss at 12, 18 and 24 months postpartum [11]. In the KAN-DO postpartum nutrition intervention study with 308 women who were overweight/obese [12], only energy intake at baseline (2–7 months postpartum) was a significant negative predictor of weight change at 12–17 month follow-up. A cluster-randomized controlled trial of 2280 women with GDM randomized to usual care or lifestyle (diet + physical activity) perinatal intervention found that the intervention group retained less weight at 6 months postpartum than the usual care group, but the weight retention was markedly attenuated at 12 months postpartum [13]. All of the above intervention studies are fairly non-specific and target a variety of healthful dietary and/or physical activity behaviors.

The purpose of this study was to evaluate if the intake of specific food and beverages at 6–9 weeks postpartum predicts SPPWR at 1-year in women after GDM pregnancy. We hypothesized that women who consume high amounts of high fat foods (such as processed meats, butter and fried foods), regular soda, sweet grains, and low amounts of dietary fiber, low-fat dairy, lean meats, whole grains, fruit and vegetables at 6–9 weeks postpartum will have higher odds of SPPWR at 1-year postpartum.

## Methods

### Study participants and setting

Data is from the Study of Women, Infant Feeding, and Type 2 Diabetes After GDM Pregnancy (SWIFT), which is a prospective, observational cohort of 1035 women with GDM pregnancy who delivered a singleton, live birth ≥35 weeks of gestation, 20–45 years, and no history of diabetes [14]. SWIFT women were recruited from 12 Kaiser Permanente medical centers and medical office facilities from 2008 to 2011 throughout the 5000 mile^2^ northern California region. Women were diagnosed with GDM between 24 and 34 weeks gestation using the Carpenter and Coustan critera [15]. The complete description of the methods and main outcomes of the study have been previously reported [14, 16]. The Kaiser Permanente Institutional Review Board approved this study protocol. This current analysis only uses data collected during pregnancy, and at study baseline (6–9 weeks postpartum) and 1-year postpartum.

### Sample selection

Of 1035 women in the SWIFT Study, 21 were excluded due to elevated fasting glucose ≥126 mg/dL, or 2-h glucose ≥200 mg/dL at 6–9 weeks postpartum diagnostic of diabetes, two were ineligible with an infant born <35 weeks gestation, and two women dropped out at baseline. We also excluded 28 women missing beverage intake at baseline, 65 women missing 1-year postpartum body weight measurements, and 29 women missing dietary intake at baseline or glucose tolerance status at 1-year follow up. Therefore, this analysis included 888 women.

### Overview of data collection

SWIFT participants provided written informed consent to participate in three in-person visits at 6–9 weeks (baseline), 1-year and 2-years postpartum [14]. At each study visit, trained research staff administered the 2-h 75 g oral glucose tolerance tests (OGTT), measured weight and height, and administered surveys to collect information on dietary intake and other behaviors, socio-demographics, infant feeding practices and other attributes. Both interviewer and self-administered questionnaires collected information on socio-demographics, age, race/ethnicity, family history of diabetes, previous GDM diagnosis, enrollment in special supplemental nutrition program for Women, Infants, and Children (WIC), medical history, and previous contraception.

### Clinical perinatal maternal and infant data

Prenatal and delivery characteristics were obtained from electronic medical records (EMR) that included prenatal laboratory results and dates of GDM diagnosis from 3-h 100 g oral OGTTs, pre-pregnancy weight, weight at delivery, pregnancy complications, and type of GDM treatment. Gestational weight gain (kg) was defined as the last weight before delivery minus reported pre-pregnancy weight [14]. The EMR also provided data on newborn birth weight, length, and gestational age.

### Anthropometry

Pre-pregnancy weight was self-reported and validated with measurements within one year prior to pregnancy, and first trimester measured weights. At 6–9 weeks and 1-year postpartum women were weighed on a calibrated digital scale (Tanita, Model WB110A, 100A) to the nearest 0.1 lb. in light clothing. Height was measured in bare feet to the nearest centimeter using a stadiometer (Seca, Model 69,072) [14]. Gestational weight gain (GWG) categories (within or below vs. excessive) for pre-pregnancy BMI were determined by the Institute of Medicine (IOM) [17]. Postpartum weight retention was calculated by subtracting the pre-pregnancy weight from weight at 1-year postpartum. Participants were categorized by SPPWR defined as ≥5 kg retained, or not SPPWR defined as <5 kg retained at 1-year postpartum [18].

### Infant feeding

Infant feeding practices, including breastfeeding intensity and duration, and formula feeding were assessed prospectively from delivery via feeding diaries, telephone calls at 1 month post-delivery, and monthly mailed questionnaires. Women were asked to report number of breast-milk and/or formula feeding episodes per 24-h, quantity of formula per feeding, and total number of feedings per day from birth to one month, and the average of these same frequencies within the past week and return the surveys each month. At study visits, research staff also administered questionnaires to assess infant feeding practices during baseline (6–9 weeks) and 1-year postpartum in-person visits [14]. For these analyses, breastfeeding duration was used as a covariate in the models.

### Oral glucose tolerance test

Two-hour 75 g OGTTs were performed at the baseline exam (6–9 weeks postpartum) and at follow-up exams at 1-year postpartum. Fasting and 2-h plasma samples were stored at −70 °C until assayed. Plasma glucose was analyzed enzymatically with the Hitachi 917 Autoanalyzer by the University of Washington, Northwest Lipid Metabolism and Diabetes Research Laboratory. The Diabetes Endocrinology Research Center Immunoassay Core Laboratory performed the assay of total immunoreactive insulin (microunits/mL) using a double-antibody radioimmunoassay with high precision.

### Glucose tolerance classification

Glucose tolerance was defined as normal, glucose intolerant [e.g., prediabetes (Pre-DM) defined as impaired fasting glucose (IFG) between 100 and 125 mg/dL, and impaired glucose tolerance (IGT) for 2-h 75 g post glucose between 140 and 199 mg/dL, or diabetes (DM) based on the American Diabetes Association diagnostic criteria for the 2-h 75 g OGTT (fasting ≥126 mg/dL and/or 2-h ≥ 200 mg/dL) and a repeat OGTT for women with elevated values [19]. The transition in glucose tolerance from baseline to 1-year postpartum was classified into one of three groups: Normal at both baseline and 1-year follow-up (*n* = 376); Pre-diabetes Pre-DM at baseline and no diabetes (DM at 1-year (*n* = 261); and progression from normal tolerance to prediabetes (Pre-DM) or DM at 1-year (*n* = 257).

### Maternal dietary intake and physical activity

At 6–9 weeks, women completed the 18-item PrimeScreen to assess dietary intake of total energy (kcal/d), total and animal fat (% of kcals), dietary fiber (% of kcals), dark leafy green vegetables, cruciferous vegetables (broccoli), carrots, other vegetables, citrus fruits, fruits, sweet grains, whole grains, whole and low-fat dairy products, pasta/rice/noodles, whole eggs, margarine, high fat and lean meats, and fried foods. The PrimeScreen was reduced from 135 items to 18 items, and includes the most frequently consumed foods based on data from the Nurses Health Study [20] and the Health Professional study [21]. The 18-item PrimeScreen was validated against the 131-item semi-quantitative food frequency questionnaire (SSFQ) and plasma levels of selected nutrients in a sample of 160 multi-ethnic men and women [22]. Participants completed the PrimeScreen twice, two to four weeks apart, and reproducibility correlation coefficients were moderate to strong, ranging from 0.50 to 0.87. The correlation coefficients between the PrimeScreen and the SFFQ were weak for dark leafy vegetables (0.44), sweet grains (0.48), moderate for dietary fiber (0.58), saturated fat (0.59), animal fat (0.55), cruciferous vegetables (0.64), fruits (0.58), citrus fruits (0.61), whole milk dairy (0.71), margarine (0.67), whole grains (0.51), pasta, rice, and noodles (0.51), beef, pork or lamb 0.63), and fried foods (0.69), and strong for carrots (0.70), low-fat dairy (0.71), eggs (0.82), and processed meats (0.74). The PrimeScreen did not include questions on SSB intake, thus the 13-item Caffeine Questionnaire, developed by Fred Hutchinson Cancer Center was also administered. This questionnaire identified frequency of regular soda (with and without caffeine) and diet soda (with and without caffeine) intake at 6–9 weeks postpartum. This questionnaire has been used to assess beverage consumption in other adult populations [23]. The frequency of consumption was reported in nine categories ranging from “never or less than once per month” to “six or more per day”. A medium serving size was defined as a 12-oz can of soda, and based on these guidelines, women reported their usual serving size per day as small, medium, or large. However, given the rather small percentage of women reporting daily intake of SSB intake, the responses were collapsed into the following categories: never, 1–3 servings per month, 1 serving per week, and 2–4+ servings per week. Dietary intake from the PrimeScreen and the Caffeine questionnaire were analyzed separately.

Physical activity was measured at baseline and 1-year postpartum with the Pregnancy Physical Activity Questionnaire, which is a 32-item, semi-quantitative, validated questionnaire (reproducibility measures of 0.78 to 0.93) that was adapted for the postpartum period [14, 24].

### Statistical analyses

Differences in participant characteristics, dietary intake, and physical activity variables between postpartum weight retention categories were assessed using chi-square tests for categorical variables (maternal race, GWG groups, glucose tolerance groups at baseline, breastfeeding intensity and duration categories, glucose tolerance transition groups, and all food groups) and analysis of variance (ANOVA) with F-tests for continuous variables (maternal age, pre-pregnancy BMI, GWG, breastfeeding duration, infant gestation age, infant birth weight, energy intake, dietary fat, fiber and glycemic index).

Variables associated with postpartum weight retention groups, were evaluated via logistic regression models to estimate odds of substantial postpartum weight retention including variables for food and beverage groups. The referent category used was the “healthy” dietary profile (e.g., never ate eggs, processed meats, fried foods, or never drank soda at baseline). Covariates evaluated as potential confounders based on a priori hypotheses included BF duration, maternal education level, pre-pregnancy BMI, gestational weight gain, birth weight, energy intake, glucose tolerance categories at baseline and transition of glucose tolerance categories (prediabetes or diabetes) from baseline to 1-year postpartum. Next, all food and beverage variables significantly associated with SPPWR in the bivariate chi-square/ANOVA analysis (*P* < 0.05) were included as covariates in the logistic regression models. SAS for Windows 9.1.3 (SAS Institute Inc., Cary, NC) was used, with significance level set at *P* < 0.05.

## Results

Characteristics for the total sample and according to 1-year PPWR categories are shown in Table 1. Women with substantial PPWR (weight retention ≥5 kg, *n* = 690) at 1-year postpartum compared to those women with normal PPWR (weight retention <5 kg, *n* = 198) were younger, Non-Hispanic White (NHW), Non-Hispanic Black (NHB), or Hispanic, had lower education levels, were more likely to receive WIC benefits, had higher pre-pregnancy BMI, increased GWG, higher prevalence of pre-diabetes at baseline, increased transition of pre-diabetes or T2D at 1-year, and had decreased breastfeeding durations (*P* < 0.02).

**Table 1:** Prenatal and postpartum characteristics of the women with GDM enrolled into the SWIFT Study (*N* = 888)

Characteristics	Total *N* = 888	< 5 kg 1-yr. weight retention (*N* = 690)	≥ 5 kg 1-yr. weight retention (*N* = 198)	*P*-value
Maternal Age (yr)	32.6 ± 4.8	32.9 ± 4.6	31.8 ± 5.1	0.005
Maternal Race/ Ethnicity				0.005
Non-Hispanic White	214 (24.1)	162 (23.5)	52 (26.3)	
Non-Hispanic Black	68 (7.7)	44 (6.4)	24 (12.1)	
Hispanics	260 (29.3)	197 (28.6)	63 (31.8)	
Asian	329 (37.1)	275 (39.9)	54 (27.3)	
Other	17 (1.9)	12 (1.7)	5 (2.5)	
Education level				<.001
High school or less	207 (23.3)	155 (22.5)	52 (26.2)	
Some college	245 (27.6)	172 (24.9)	73 (36.9)	
≥ 4 years of college	436 (49.1)	363 (52.6)	73 (36.9)	
Receiving WIC benefits				<.001
No	668 (75.2)	538 (78.0)	130 (65.7)	
Yes	220 (24.8)	152 (22.0)	68 (34.3)	
Maternal Pre-pregnancy BMI (kg/m^2^)	29.4 ± 7.1	28.9 ± 6.7	31.1 ± 8.3	<.001
Total GWG (kg)	10.2 ± 6.6	9.2 ± 5.9	14.0 ± 7.4	<.001
Weight Retention at Baseline (kg)	1.2 ± 6.0	0.1 ± 5.4	5.3 ± 6.0	<.001
Breastfeeding Duration (months)	8.9 ± 7.2	9.3 ± 7.2	7.8 ± 7.2	0.001
Infant Gestational Age (weeks)	39.0 ± 1.1	39.1 ± 1.1	39.0 ± 1.2	0.608
Infant Birth Weight (g)	3404.6 ± 500.8	3392.4 ± 494.0	3446.8 ± 522.7	0.178
Total GWG for IOM Levels:				<.001
Excessive	306 (34.5)	188 (27.2)	118 (59.5)	
Within or Below	582 (65.5)	502 (72.8)	80 (40.5)	
2-h 75 g OGTT at Baseline:				0.002
Normal	579 (65.2)	468 (67.8)	111 (56.1)	
Pre-diabetes	309 (34.8)	222 (32.2)	87 (43.9)	
Breastfeeding Duration Categories:				0.01
0 to <2 months	169 (19.0)	123 (17.8)	46 (23.2)	
2 to 5 months	175 (19.7)	126 (18.3)	49 (24.8)	
> 5 to 10 months	186 (21.0)	144 (20.9)	42 (21.2)	
> 10 months	358 (40.3)	297 (43.0)	61 (30.8)	
Transition to Pre-DM or DM at 1-yr.:				0.004
Normal at baseline and 1-yr	361 (42.4)	301 (45.3)	60 (31.9)	
Pre-DM at baseline, & not DM at 1-yr	255 (30)	187 (28.2)	68 (36.2)	
Normal at baseline, developed pre-DM or DM at 1-yr	236 (27.7)	176 (26.5)	60 (31.9)	

Data are n (%), or mean ± SD; Chi-square or t-tests were performed to assess significant different in demographics between weight retention categories. *BMI* Body Mass Index, *WIC* Women, Infant, and Children, *GWG* gestational weight gain, *IOM* Institute of Medicine, *OGTT* oral glucose tolerance test, *BF* breastfeeding, *Pre-DM* Pre-diabetes, *DM* Diabetes

Dietary intake and physical activity variables for the whole sample and between the PPWR categories are shown in Table 2. Using chi-square tests, the following diet variables differed by PPWR categories: whole eggs (*P* = 0.03), processed meats (*P* = 0.03), fried foods (*P* = 0.0005), and soda intake per day (*P* = 0.005).

**Table 2:** Dietary intake variables [mean ± /sd or n (%)] at baseline (6–9 week postpartum) by 1-year postpartum weight retention categories (≥ 5 kg or <5 kg) among women with recent GDM (*N* = 888)^a,b^

Dietary Intake from PrimeScreen At 6–9 weeks postpartum (baseline)	Total	< 5 kg 1 yr	≥ 5 kg 1 yr	*P*-value
		wt retention	wt retention	
	*N* = 888	(*N* = 690)	(*N* = 198)	
Nutrients				
Energy intake (kcal/d)	834.2 ± 340.9	832.8 ± 343.2	839.1 ± 333.7	0.82
Animal Fat (% of kcal)	25.2 ± 8.2	25.2 ± 8.3	25.3 ± 7.9	0.84
Saturated Fat (% of kcal)	26.8 ± 8.6	26.8 ± 8.7	27.1 ± 8.3	0.62
Fiber (g/day)	8.4 ± 3.9	8.4 ± 3.9	8.2 ± 3.6	0.60
PrimeScreen Food Groups				
Dark Leafy Green Vegetables				0.41
≥ 5 serv/wk	267 (30.1)	212 (30.7)	55 (27.8)	
2–4 serv/wk	434 (48.9)	327 (47.4)	107 (54.0)	
1 serv/wk	119 (13.4)	94 (13.6)	25 (12.6)	
None	65 (7.3)	55 (8.0)	10 (5.1)	
Broccoli, cauliflower, cabbage				0.65
≥ 5 serv/wk	80 (9.0)	61 (8.8)	19 (9.6)	
2–4 serv/wk	346 (39.0)	278 (40.3)	68 (34.3)	
1 serv/wk	242 (27.3)	183 (26.5)	59 (29.8)	
None	217 (24.4)	166 (24.1)	51 (25.8)	
Carrots				0.93
≥ 5 serv/wk	88 (9.9)	70 (10.1)	18 (9.1)	
2–4 serv/wk	322 (36.3)	250 (36.2)	72 (36.4)	
1 serv/wk	239 (26.9)	183 (26.5)	56 (28.3)	
None	233 (26.2)	183 (26.5)	50 (25.3)	
Other Vegetables (peas, corn, green beans)				0.59
≥ 5 serv/wk	333 (37.5)	264 (38.3)	69 (34.9)	
2–4 serv/wk	416 (46.9)	316 (45.8)	100 (50.5)	
1 serv/wk	99 (11.2)	76 (11.0)	23 (11.6)	
None	31 (3.5)	27 (3.9)	4 (2.0)	
Citrus fruits (including juice)				0.30
≥ 5 serv/wk	174 (19.6)	127 (18.4)	47 (23.7)	
2–4 serv/wk	297 (33.5)	227 (32.9)	70 (35.4)	
1 serv/wk	162 (18.2)	133 (19.3)	29 (14.7)	
None	250 (28.2)	199 (28.8)	51 (25.8)	
Fruits (apples, pears, bananas, grapes)				0.44
≥ 5 serv/wk	371 (41.8)	286 (32.2)	85 (42.9)	
2–4 serv/wk	371 (41.8)	291 (32.8)	80 (40.4)	
1 serv/wk	89 (10.0)	65 (7.3)	24 (12.1)	
None	57 (6.4)	48 (5.4)	9 (4.6)	
Sweet grains (cookies, muffins, cakes)				0.63
≥ 5 serv/wk	108 (12.2)	85 (12.3)	23 (11.6)	
2–4 serv/wk	330 (37.2)	263 (38.1)	67 (33.8)	
1 serv/wk	255 (28.7)	192 (27.8)	63 (31.8)	
None	193 (21.7)	149 (21.6)	44 (22.2)	
Whole grains (whole grain bread, brown rice)				0.52
≥ 5 serv/wk	423 (47.6)	322 (46.7)	101 (51.0)	
2–4 serv/wk	290 (32.7)	233 (33.8)	57 (28.8)	
1 serv/wk	97 (10.9)	73 (10.6)	24 (12.1)	
None	78 (8.8)	62 (9.0)	16 (8.1)	
Pasta, rice, noodles				0.60
≥ 5 serv/wk	256 (28.8)	203 (29.4)	53 (26.8)	
2–4 serv/wk	406 (45.7)	306 (44.4)	100 (50.5)	
1 serv/wk	151 (17.0)	122 (17.7)	29 (14.7)	
None	72 (8.1)	57 (8.3)	15 (7.6)	
Whole milk dairy foods (whole milk, hard cheese, butter, ice cream)				0.25
≥ 5 serv/wk	349 (39.3)	259 (37.5)	90 (45.5)	
2–4 serv/wk	309 (34.8)	243 (35.2)	66 (33.3)	
1 serv/wk	103 (11.6)	82 (11.9)	21 (10.6)	
None	126 (14.2)	105 (15.2)	21 (10.6)	
Whole eggs				0.031
≥ 5 serv/wk	178 (20.1)	148 (21.5)	30 (15.2)	
2–4 serv/wk	355 (40.0)	284 (41.2)	71 (35.9)	
1 serv/wk	196 (22.1)	141 (20.4)	55 (27.8)	
None	156 (17.6)	114 (16.5)	42 (21.2)	
Margarine				0.75
≥ 5 serv/wk	55 (6.2)	40 (5.8)	15 (7.6)	
2–4 serv/wk	96 (10.8)	74 (10.7)	22 (11.1)	
1 serv/wk	125 (14.1)	94 (13.6)	31 (15.7)	
None	609 (68.6)	480 (69.6)	129 (65.2)	
Low-fat milk products (low-fat/skim milk, yogurt, cottage cheese)				0.81
≥ 5 serv/wk	381 (42.9)	299 (43.3)	82 (41.4)	
2–4 serv/wk	233 (26.2)	185 (26.8)	48 (24.2)	
1 serv/wk	92 (10.4)	70 (10.1)	22 (11.1)	
None	178 (20.1)	133 (19.3)	45 (22.7)	
Fish/Seafood (not fried, but broiled, baked)				0.71
≥ 5 serv/wk	35 (3.9)	30 (4.4)	5 (2.5)	
2–4 serv/wk	204 (23.0)	158 (22.9)	46 (23.2)	
1 serv/wk	263 (29.6)	204 (29.6)	59 (29.8)	
None	386 (43.5)	298 (43.2)	88 (44.4)	
Processed meats (sausages, salami, bologna, hot dogs, bacon)				0.027
≥ 5 serv/wk	47 (5.3)	28 (4.1)	19 (9.6)	
2–4 serv/wk	248 (27.9)	200 (29.0)	48 (24.2)	
1 serv/wk	246 (27.7)	189 (27.4)	57 (28.8)	
None	345 (38.9)	27 (39.3)	74 (37.4)	
Beef, pork, or lamb				0.85
≥ 5 serv/wk	175 (19.7)	135 (19.6)	40 (20.2)	
2–4 serv/wk	397 (44.7)	309 (44.8)	88 (44.4)	
1 serv/wk	150 (16.9)	114 (16.5)	36 (18.2)	
None	163 (18.4)	129 (18.7)	34 (17.2)	
Fried foods (fried chicken, fish, seafood, French fries, onion rings)				<.001
≥ 5 serv/wk	32 (3.6)	18 (2.6)	14 (7.1)	
2–4 serv/wk	208 (23.4)	147 (21.3)	61 (30.8)	
1 serv/wk	353 (39.8)	281 (40.7)	72 (36.4)	
None	295 (33.2)	244 (35.4)	51 (25.8)	
Dietary Variables obtained from Caffeine Screener at Baseline				
Cola Soda				<0.001
≥ 2 serv/wk	93 (13.5)	49 (24.8)	142 (16.0)	
1 or less serv/wk	189 (27.4)	56 (28.3)	245 (27.6)	
None	408 (59.1)	93 (47.0)	501 (56.4)	
Diet Cola Soda				0.37
≥ 2 serv/wk	96 (13.9)	34 (17.2)	130 (14.6)	
1 or less serv/wk	102 (14.8)	27 (13.6)	129 (14.5)	
None	488 (70.7)	134 (67.7)	622 (70.1)	
Physical Activity (PA) Score				
Total PA, *met hr./wk*	46.1 ± 21.1	46.0 ± 21.1	46.4 ± 21.1	0.80
Moderate to vigorous PA, *met hr./wk*	24.7 ± 15.4	24.6 ± 15.4	25.0 ± 15.4	0.72

^a^Data are n (%), or mean ± SD

^b^ANOVA (for continuous dependent variables) and chi-square (for categorical dependent variables) were run to assess differences in food/beverage variables between weight retention categories. Met hr./wk. = *metabolic equivalent hour per week*

Given the above diet variables differed by SPPWR, logistic regression analyses were run to further investigate the differences. Unadjusted and adjusted odds ratios for soda and fried food consumption at baseline in predicting SPPWR (≥5 kg) at 1-year postpartum for the total sample are shown in Table 3. Mothers who reported drinking soda ≥2 servings per week (*n* = 142) compared to those who reported never drinking regular soda (*n* = 501) at baseline had a 2-fold higher odds of SPPWR (OR = 2.31, 95% CI:1.53–3.49, *P* < 0.001). These results remained (OR = 1.95, 95% CI:1.22–3.11, *P* = 0.005) after controlling for *a prior* covariates (i.e., BF duration, pre-pregnancy BMI, education level, gestational weight gain, and glucose tolerance transition). However, the odds ratio was attenuated further after adjustment for other food groups (OR = 1.61, 95% CI: 0.98–2.66, *P* = 0.06).

**Table 3:** Odds Ratios for soda consumption categories/fried food consumption categories at 6–9 weeks postpartum and substantial postpartum weight retention (≥5 kg) at 1-year for entire sample^a^

Total sample	Soda consumption categories				
*N* = 888	None (*n* = 501)	1 or less serv/wk. (*n* = 245)	≥ 2 serv/wk. (*n* = 142)		*Overall Groups P*-value
Model 1:					
Unadjusted	Referent	1.30 (0.90–1.89)	2.31 (1.53–3.49)*		0.005
Model 2:^b^					
Adjusted	Referent	1.04 (0.69–1.58)	1.94 (1.22–3.07)*		0.014
Model 3:^c^					
Adjusted	Referent	1.04 (0.68–1.57)	1.95 (1.22–3.11)*		0.014
Model 4:^d^					
Adjusted	Referent	1.00 (0.65–1.53)	1.61 (0.98–2.66)^†^		0.136
Fried food consumption categories					
*N* = 888	None (*n* = 295)	1 serv/wk. (*n* = 353)	2–4 serv/wk. (*n* = 208)	≥ 5 serv/wk. (*n* = 32)	*Overall Groups P*-value
Model 1:					
Unadjusted	Referent	1.23 (0.82–1.82)	1.99 (1.30–3.04)*	3.72 (1.74–7.96)*	<0.001
Model 2:^b^					
Adjusted	Referent	1.06 (0.69–1.63)	1.84 (1.15–2.92)*	3.38 (1.44–7.96)*	0.003
Model 3:^c^					
Adjusted	Referent	1.12 (0.72–1.73)	1.89 (1.18–3.02)*	3.50 (1.48–8.29)*	0.003
Model 4:^e^					
Adjusted	Referent	1.12 (0.71–1.75)	1.80 (1.08–3.00)*	3.38 (1.36–8.38)*	0.015

*Statistically significant associations (*p* < 0.01 or <0.05) for individual group compared to the referent group

^†^Borderline statistically significant association (*p* = 0.06) for individual group compared to the referent group

^a^Data are OR (95% CI)

^b^Model 2 adjusted for BF duration, pre-pregnancy BMI, education level, gestational weight gain

^c^Model 3 adjusted for BF duration, pre-pregnancy BMI, education level, gestational weight gain, (baseline glucose tolerance plus transition of glucose tolerant categories from baseline (6–9 weeks postpartum) to 1-year postpartum)

^d^Model 4 adjusted for BF duration, pre-pregnancy BMI, education level, gestational weight gain, (baseline glucose tolerance plus transition of glucose tolerant categories from baseline (6–9 weeks postpartum) to 1-year postpartum) and fried foods, eggs, and processed meats

^e^Model 4 adjusted for BF duration, pre-pregnancy BMI, education level, gestational weight gain, (baseline glucose tolerance plus transition of glucose tolerant categories from baseline (6–9 weeks postpartum) to 1-year postpartum) and soda, eggs, and processed meats

Eating fried foods was associated with higher odds of SPPWR in unadjusted and adjusted models. Consuming ≥5 servings of fried food a week (*n* = 32) versus none (*n* = 295) was associated with a threefold higher odds of SPPWR (OR = 3.72, 95% CI:1.74–7.96, *P* < 0.001), and consuming 2–4 servings per week of fried foods versus none was associated with a two-fold greater odds of SPPWR (OR = 1.99, 95% CI:1.30–3.03, *P* = 0.002). These results for fried foods remained highly significant after adjusting for other food and beverage intake and all a priori covariates (OR = 3.38, 95% CI:3.38–1.36, *P* = 0.009 for intake *o*f ≥ 5 serv/wk., and OR = 1.80, 95% CI:1.8–1.08, *P* = 0.02 for intake of 2–4 serv/wk. versus none).

Unadjusted and adjusted odds ratios for whole eggs and processed meat consumption at baseline predicting SPPWR (≥5 kg) at 1-year postpartum for the total sample are shown in Table 4. Women who reported eating ≥5 servings of eggs a week (*n* = 178) had a 45% lower unadjusted odds of SPPWR (OR = 0.55, 95% CI:0.32–0.93, *P* = 0.027), but the association was no longer statistically significant after accounting for all *a prior* covariates and other food and beverage intake (OR = 0.69, 95% CI:0.37–1.29, *P* = 0.24). Women who reported eating ≥5 servings of processed meats per week (*n* = 47) had a 2-fold higher odds of SPPWR (OR = 2.48, 95% CI:1.31–4.68, *P* = 0.005), but, the association was no longer statistically significant after adjustment for *a prior* covariates and other food and beverage intake (OR = 1.46, 95% CI:0.67–3.20, *P* = 0.34).

**Table 4:** Odds Ratios for Whole Egg categories and Process Meat consumption categories at 6–9 weeks postpartum and substantial postpartum weight retention (≥5 kg) at 1-year (entire sample)^a^

Whole egg categories					
*N* = 883	None (*n* = 156)	1 serv/wk. (*n* = 195)	2–4 serv/wk. (*n* = 354)	≥ 5 serv/wk. (*n* = 178)	*Overall Groups P*-value
Model 1:					
Unadjusted	Referent	1.07 (0.67–1.71)	0.68 (0.44–1.06)	0.55 (0.32–0.93)*	0.021
Model 2:^b^					
Adjusted	Referent	1.26 (0.73–2.17)	0.71 (0.43–1.18)	0.65 (0.36–1.19)	0.043
Model 3:^c^					
Adjusted	Referent	1.38 (0.81–2.36)	0.79 (0.48–1.29)	0.74 (0.41–1.34)	0.058
Model 4:^d^					
Adjusted	Referent	1.35 (0.78–2.36)	0.78 (0.46–1.32)	0.69 (0.37–1.29)	0.064
Processed meats Categories					
*N* = 883	None (*n* = 344)	1 serv/wk. (*n* = 245)	2–4 serv/wk. (*n* = 247)	≥ 5 serv/wk. (*n* = 47)	*Overall Groups P*-value
Model 1:					
Unadjusted	Referent	1.11 (0.75–1.64)	0.88 (0.59–1.32)	2.48 (1.31–4.68)*	0.021
Model 2:^b^					
Adjusted	Referent	0.90 (0.58–1.40)	0.65 (0.41–1.05)	1.28 (0.60–2.72)	0.199
Model 3:^c^					
Adjusted	Referent	0.92 (0.59–1.44)	0.83 (0.53–1.31)	1.78 (0.87–3.63)	0.238
Model 4:^e^					
Adjusted	Referent	0.91 (0.57–1.44)	0.69 (0.42–1.13)	1.46 (0.67–3.20)	0.202

^*^statistically significant associations (*p* < 0.05) for individual group comparison to the referent group

^a^Data are OR (95% CI)

^b^Model 2 adjusted for BF duration, pre-pregnancy BMI, education level, gestational weight gain

^c^Model 3 adjusted for BF duration, pre-pregnancy BMI, education level, gestational weight gain, (baseline glucose tolerance plus transition of glucose tolerant categories from baseline (6–9 weeks postpartum) to 1-year postpartum)

^d^Model 4 adjusted for BF duration, pre-pregnancy BMI, education level, gestational weight gain, (baseline glucose tolerance plus transition of glucose tolerant categories from baseline (6–9 weeks postpartum) to 1-year postpartum) and soda, fried foods, and processed meats

^e^Model 4 adjusted for BF duration, pre-pregnancy BMI, education level, gestational weight gain, (baseline glucose tolerance plus transition of glucose tolerant categories from baseline (6–9 weeks postpartum) to 1-year postpartum) and soda, fried foods and eggs

## Discussion

This study found that higher intakes of soda, and fried food intake during early postpartum periods were independently associated with substantial postpartum weight retention. However, processed meats and egg intake showed no independent association after controlling for other food and beverage intake. To our knowledge, this is the first study to show direct associations for high intakes of fried food and soda intake with SPPWR in a high-risk GDM population.

Fried food intake at baseline was linked to three-fold higher odds of SPPWR and this impact remained highly significant after controlling for soda intake and other confounding variables. National data shows that approximately 31% of adults consume fast food on any given day and fast food intake is associated with an increase in daily energy intake of 190 kcals per day and over 10 additional grams of fat [25]. In a cross-sectional study of 226 women with GDM by Zehle et al., 26% of the women consumed fried food at least twice per week [26]. Similarly, we found that 27% of our sample consumed fried food at least twice per week. Numerous adult studies have consistently found a link between fast food intake and obesity, [27–29] however, few studies have examined the relationship of fast food and/or fried foods on postpartum weight retention. In the Active Mothers Postpartum study, which included 450 overweight or obese White and African American postpartum women, lower junk food intake (which included French fries, fast food, chips, and soda and sugar sweetened beverages) at baseline (6 weeks postpartum) was linked to greater weight loss at 12-, 18, and 24-months postpartum [11]. These findings along with our findings, suggests that targeting the reduction of fried foods early in postpartum may have profound impacts on reducing PPWR.

Strong evidence exists to support that sugar sweetened beverage (SSB) intake, including soda intake, is a leading culprit in both pediatric and adult obesity [30, 31]. National data in 2009–2010 found that US adults consumed an estimated average 151 kcal/day of SSB, with regular soda and fruit drinks representing the leading sources of SSB energy intake [32]. National data showed that 26% of adults consumed regular soda or fruit drinks or both at least once daily and consumption was highest among adults between 18 and 34 years of age and in minority adults [33]. Fewer studies have examined SSB intake during pregnancy or postpartum periods. In a cross-sectional study with 115 lactating Mexican women living in Mexico City, 23% of women drank approximately 2.5 servings of SSB per day, in the first three months postpartum [34]. In the current study, reported soda intake was significantly lower than the above study and the national average, where only 23% of GDM women drank soda 2–4+ servings per week. Regardless of the lower intake, soda intake during early postpartum periods was linked to increased rates of SPPWR. This data suggests that even low to moderate soda intake (i.e., 2–4+ servings per week) can hinder postpartum weight loss.

However, the link between soda consumption and SPPWR was attenuated somewhat after controlling for processed meats, and egg consumption. While research consistently shows that increased fast food intake is linked to increases in SSB intake [25, 29], our findings suggest that fried food intake compared to soda intake has a more profound impact on SPPWR. One possible explanation for this finding is that these GDM women were often counseled by their healthcare team to limit sugar intake, but fried food intake may not have been addressed.

In the current study, processed meat intake and egg intake were not associated with odds of SPPWR after accounting for covariates, fried foods and regular soda. It is possible that some processed meats were also counted as fried foods, for example bacon and sausage, but fried foods in total had a stronger impact on weight gain. Thus, the fried foods intake may overwhelm any impact of processed meats on SPPWR. Another explanation is that subjects who consumed fried foods, also consumed higher quantities of processed foods and lower quantities of eggs, but fried foods had a stronger effect on PPWR than the other high fat foods.

Recently a number of RCT have been conducted with GDM women targeting postpartum weight reduction. A feasibility RCT conducted by Ferrara et al. [35] with 197 women with GDM found no significant differences in postpartum weight loss between those women receiving an intensive telephone-based lifestyle intervention, which targeted increases in moderate intensity physical activity, exclusive breastfeeding for six months, and reductions in caloric and fat intake, compared to the control group. However, the intervention appeared to be more effective among women who did not exceed the guidelines for GWG. A cluster-RCT conducted by Ferrara et al. [13] including 2280 women with GDM found that a similar telephone based lifestyle intervention compared to usual care group resulted in approximately one kilogram less weight retention at 6 months postpartum, but the weight retention was attenuated at 12 months postpartum. A 12-week lifestyle intervention conducted by Bertz et al. [36] with 61 Swedish women who are overweight/obese, found that women who decreased their energy intake, primarily by decreasing carbohydrates, sucrose and fat intake at 12 weeks and sustained these reductions at 1-year postpartum had significant reductions in weight loss at both 12 weeks and 1-year. Another RCT with 75 women with GDM found that a 1-year web-based lifestyle intervention that began at 6 weeks postpartum decreased postpartum weight retention at 1-year [37]. To date, the interventions targeting postpartum weight reduction in GDM women have primarily used lifestyle interventions that target multiple dietary and physical activity behaviors.

Evidence remains unclear on whether intervention targeting multiple behavior changes vs. single behavior change is more effective at weight loss. A recent review of 22 meta-analyses studies, showed that interventions targeting a single health behavior change (i.e., nutrition vs. physical activity) were more effective at improving the targeted behavior, while multiple health behavior interventions (i.e., nutrition + physical activity) resulted in greater weight loss [38]. However, this review classified the single health behavior interventions as either nutrition or physical activity behaviors, which ultimately still includes multiple behavior targets within each category. Few studies have examined whether or not changes in a single dietary behavior could have more weight loss effects compared to changing multiple dietary behaviors. More long-term interventions targeting single or few dietary behavior changes are warranted to fully understand the long-term benefits of such an approach.

Focusing on one dietary component, such as decreasing fried food intake, is a very concise and easy message to focus on, and might be easier for postpartum women with GDM to understand and achieve. Individuals asked to make multiple behavior changes report feeling confused and overwhelmed [39] and this may push them to quit and return to their unhealthy lifestyles. Particularly, because new mothers are facing a variety of stressors, including sleep deprivation, hormonal changes, and the demands of caring for a newborn [40]. In addition, body dissatisfaction and depression increases significantly during postpartum periods [41, 42]. Thus, a simpler and more succinct intervention to follow, such as decreasing soda intake, may be easier and more attainable for new mothers.

Contrary to our hypothesis, low intakes of whole grains, fruit and vegetables were not linked to SPPWR. In a large national sample, women with self-reported GDM who lived with their children compared to their counterparts without GDM were significantly less likely to meet the five or more servings of fruit and vegetable recommendations [9]. In another cross-sectional study of 226 women with GDM, only 5% consumed the five or more servings a day of vegetables and 44% consumed two or more servings a day of fruit. Higher intakes of fruit, vegetables, and whole grains have been shown to reduce risk of obesity and associated chronic diseases in adults [43–45], however, few studies have examined how these specific foods are related to postpartum weight retention. One retrospective study with 444 Swedish women showed no difference in fruit and vegetable intake during pregnancy between women with a history of normal pregnancy compared to those with a history of GDM [46]. A possible explanation for our null findings with fruit and vegetable intake, is that the PrimeScreen survey was not designed or validated to evaluate the combined fruit and vegetable intake, and it is possible that the sum of vegetables and/or fruits would have been linked to reductions in PPWR. In addition, energy, saturated fat, animal fat and fiber intake were not associated with SPPWR, and this also might be because the PrimeScreen is less accurate in assessing energy and nutrient intake.

There are some other limitations to consider for the current study. One limitation is that the caffeine questionnaire did not include questions for other SSBs, such as juice, fruit drinks and sports drinks, which are also known to be large contributors to added sugar intake in the American diet. Another limitation is the rather small sample size of subjects consuming fried food ≥5 servings a week at baseline (*n* = 32), however, at least 23% of the population (*n* = 208) consumed fried food 2–4 times per week at baseline and this group compared to the never group also had SPPWR. There was also a large percent of women (48%) in this cohort that reported not consuming SSBs at baseline. This is likely because clinicians treating these GDM women advised them not to consume SSB intake to control their blood sugars during pregnancy, and this abstinence may have continued into early postpartum periods. Another limitation is that dietary intake may have changed from baseline to 1-year postpartum. Lastly, this was not an intervention study, so causality could not directly be addressed. However, the precise longitudinal measures obtained at baseline and 1-year postpartum allowed for models to be run assessing which dietary variables predicted postpartum weight retention.

## Conclusion

The study evaluated dietary intake and risk of weight retention among women with GDM who are seven times more likely to develop T2D later in life. Although postpartum weight loss may lower future risk of T2D [4, 5], few studies have evaluated postpartum weight retention among women with GDM, or evaluated the link between specific food and beverage intake and PPWR. Given the limited evidence on specific dietary behaviors linked to postpartum weight retention in women with GDM, these findings may be important to reduction in future diabetes risk. This study suggests that dietary modifications focused on fried food and soda reduction after delivery may lower risk of SPPWR at 1-year postpartum, particularly for women with excessive gestational weight gain. This is a very simple and straightforward message for healthcare providers to convey to women that may be an effective strategy for diabetes prevention in postpartum women with GDM.
